# *LO*ng-term follow-up after li*VE* kidney donation (LOVE) study: a longitudinal comparison study protocol

**DOI:** 10.1186/s12882-016-0227-5

**Published:** 2016-02-01

**Authors:** Shiromani Janki, Karel W. J. Klop, Hendrikus J. A. N. Kimenai, Jacqueline van de Wetering, Willem Weimar, Emma K. Massey, Abbas Dehghan, Dimitris Rizopoulos, Henry Völzke, Albert Hofman, Jan N. M. Ijzermans

**Affiliations:** Department of Surgery, Division of HPB and Transplant Surgery, Erasmus University Medical Center, ‘s-Gravendijkwal 230, 3015 CE Rotterdam, The Netherlands; Department of Internal Medicine, Division of Nephrology and Transplantation, Erasmus University Medical Center, ‘s-Gravendijkwal 230, 3015 CE Rotterdam, The Netherlands; Department of Epidemiology, Erasmus University Medical Center, ‘s-Gravendijkwal 230, 3015 CE Rotterdam, The Netherlands; Department of Biostatistics, Erasmus University Medical Center, ‘s-Gravendijkwal 230, 3015 CE Rotterdam, The Netherlands; Ernst Moritz Arndt University Greifswald, Institute for Community Medicine, Walther-Rathenau-Straße 48, D-17475 Greifswald, Germany; Department of Epidemiology, Harvard T.H. Chan School of Public Health, Boston, MA USA

**Keywords:** Living donors, Nephrectomy, Kidney transplantation, Follow-up studies, Cohort studies

## Abstract

**Background:**

The benefits of live donor kidney transplantation must be balanced against the potential harm to the donor. Well-designed prospective studies are needed to study the long-term consequences of kidney donation.

**Methods:**

The “*LO*ng-term follow-up after li*VE* kidney donation” (LOVE) study is a single center longitudinal cohort study on long-term consequences after living kidney donation. We will study individuals who have donated a kidney from 1981 through 2010 in the Erasmus University Medical Center in Rotterdam, The Netherlands. In this time period, 1092 individuals donated a kidney and contact information is available for all individuals. Each participating donor will be matched (1:4) to non-donors derived from the population-based cohort studies of the Rotterdam Study and the Study of Health in Pomerania. Matching will be based on baseline age, gender, BMI, ethnicity, kidney function, blood pressure, pre-existing co-morbidity, smoking, the use of alcohol and highest education degree. Follow-up data is collected on kidney function, kidney-related comorbidity, mortality, quality of life and psychological outcomes in all participants.

**Discussion:**

This study will provide evidence on the long-term consequences of live kidney donation for the donor compared to matched non-donors and evaluate the current donor eligibility criteria.

**Trial registration:**

Dutch Trial Register NTR3795.

## Background

Renal transplantation is the ultimate treatment for patients with end-stage renal disease (ESRD) [1]. The rise of live donor kidney transplantation has helped to narrow the gap between organ shortage and the number of ESRD patients on the transplant waiting list [2]. The benefits of live donor kidney transplantation, such as pre-emptive transplantation, superior organ quality and increased graft survival are well-known [1]. As a result live kidney donation has increased to one third of all kidney transplantations in the last decade [3]. In our transplant center over fifty percent of the kidney transplantations are performed with a kidney of a living donor. Live donor nephrectomy is performed on healthy individuals who willingly undergo major surgery for the benefit of someone else. Over the years, the discomfort of the operation has been minimalized to minimal invasive procedures in many centers with excellent quality of life [4–6].

The benefits for the recipient must be balanced against the potential harm to the donor. Studies on short-term follow-up show excellent results regarding kidney function, mortality and morbidity [7–10]. Reports have recently been published on increased long-term risk of ESRD [11–13] and increased risk of mortality following live kidney donation [14]. Studies on follow-up of ten years or more of live kidney donors demonstrate that the morbidity and mortality increases with the duration of follow-up and subsequent aging. More importantly, these long-term studies comparing donors with non-donors have contradictory outcomes regarding kidney function, incidence of hypertension, ESRD and mortality detriment to donors. The main reason for these contradictory results is the methodology of the different studies [14–22] which jeopardizes the comparability of donors and non-donors.

Furthermore, the incidence of minor co-morbidities among the general population is increasing and quickly become the standard in the potential Western donor population. This could potentially include an increase of incident co-morbidities among previous donors. The occurrence of some of these co-morbidities takes years to emerge and might be missed during a short-term follow-up. In addition, the shortage of kidney donors has led to an extension of the donor acceptance criteria in the recent years, were donors with co-morbidities such as cardiovascular disease, obesity and higher age are no longer denied for donation [23, 24]. Thus emphasizing the need for long-term follow-up of those who donated, including live kidney donors who donated with pre-existing co-morbidities.

To date most studies have merely focused on medical outcomes, resulting in a paucity of research on long-term psychological outcomes [25]. Current published studies focus mainly on the level of quality of life shortly after live kidney donation, which is high [5, 26–28]. Other outcomes, such as depression and anxiety, as well as the potential positive influence of donation on mental health, are often neglected. Therefore, an interesting question is to which extent the donation has had a positive effect on the psychological well-being of the donor.

The aim of this study is to evaluate long-term consequences after live kidney donation for the donor regarding kidney function, the incidence of hypertension, the incidence of diabetes mellitus, the incidence of fatal and non-fatal cardiovascular events, survival, quality of life and psychological well-being when compared to non-donors, and in addition within the donor population. Donors often do not make the decision to donate based on balancing risks and benefits but rather based on emotional or moral reasons. This makes it crucial to inform donors in a standardized uniform fashion when obtaining informed consent [29]. Determining the long-term impact of living donation on physical and psychological well-being is an essential part of evaluating the current donor eligibility criteria and further developing and expanding the live kidney donation program.

## Methods

### Participants and procedure - live kidney donors

The Netherlands has the highest number of live kidney donor transplantations in Europe spread over eight kidney transplant centers, with an annual living donation rate of 31.0 per million population in 2013 [30]. Over the years the Erasmus University Medical Center has developed the largest live kidney donation program of its country with approximately 140 procedures per year and is the referral center for other transplant centers in the country. In 2014, a total of 397 live kidney donor transplantations were performed in the Netherlands [31]. Thirty-five percent of these procedures were performed at Erasmus University Medical Center. All donors who donated a kidney from 1981 through 2010 at the department of Surgery of the Erasmus University Medical Center or who had their full medical work-up here prior to donation, but donated in another transplant center because of their participation in the national kidney exchange transplant program will be eligible to be included in this study. All donors were screened preoperatively by a nephrologist, transplant surgeon, anesthesiologist, and social worker and underwent imaging by renal angiography, magnetic resonance imaging or computerized tomography scan. Since 1981, data on pre-donation characteristics such as prior medical history, medication use, BMI, as well as blood and urine analysis regarding kidney function have been collected. Furthermore, consecutive donors included in two randomized controlled trials on surgical techniques between 2001–2004 and 2008–2010 [4, 32, 33] have completed questionnaires on quality of life pre-donation and at set times after the procedure, using the SF-36 questionnaire and EuroQoL questionnaire. Donors are monitored annually on their kidney function by blood- and urine analysis, blood pressure, weight and other current medical issues at the department of Nephrology of the Erasmus University Medical Center or another hospital, or by their general practitioner. Up to 2011, this database comprises 1092 live kidney donors. Approval of the Medical Ethical Committee was obtained (MEC-2012-519). Pre-donation characteristics on kidney function, BMI, pre-existing co-morbidity and medication of all donors will be updated in accordance with the hospital’s electronic patient system. Mortality will be checked in accordance with the Municipal registry. All donors who are alive will be invited by post to visit the outpatient clinic of one of three hospitals in different regions of the Netherlands for an extensive follow-up appointment, supplementary to their yearly check-up. During the study-visit the donor and study investigators will sign the consent form. During their study-visit, blood and urine will be collected for analysis and donors will undergo two interviews regarding quality of life and their psychological well-being. Furthermore, an ultrasound of their remaining kidney will be performed to measure the size. Participating donors will be reimbursed for their travel costs. Unexpected outcomes will be reported to both the donor and the treating nephrologist, who will initiate subsequent examinations and treatment if needed. The general practitioner and any other treating specialist(s) will be notified if needed. Non-responders will be contacted once again and non-participants will be asked to fill out and return a questionnaire on their medical history and quality of life. In addition, non-participants will be asked to give permission to request recent laboratory results on kidney function and new-onset co-morbidities from their medical records, in order to analyze a potential selection bias. To strengthen our results, we aim for a response of 70 percent or higher. All private data are anonymized by allocating a study number. The coordinating investigators and the principal investigator are the only ones who have access to the coding system. All data will be entered into a database, which is managed by the coordinating investigators. At the end of the study all data will be analysed together with the trial statistician (DR).

### Participants and procedure - non-donors

Non-donors will be selected from the Study of Health in Pomerania (SHIP) [34, 35] and the Rotterdam Study [36, 37], both population based cohort studies to cover the whole age range of our donors.

SHIP is a population-based cohort study initiated in 1997 among inhabitants of West Pomerania in the north-east of Germany. Two main objectives of this study were first to assess prevalence and incidence of common risk factors, subclinical disorders and clinical diseases, and second to investigate the complex associations among these [34, 35]. Data on kidney function, blood pressure, BMI, medication, incidence of cardiovascular disease and diabetes, psychological well-being and quality of life have prospectively been recorded [34]. SHIP comprises two cohorts aged 20–79 years. In our matched study, the first cohort will be used due to the longer follow-up (*n* = 4308). Multiple follow-up examinations of this first cohort were conducted between 1997 and 2015.

The Rotterdam Study is a prospective cohort study that started in 1990 in Ommoord, a neighborhood in Rotterdam, the Netherlands. The study targets cardiovascular, endocrine, hepatic, neurological, ophthalmic, psychiatric and respiratory diseases [36, 37]. As of 2008, 14,926 subjects aged 45 years or over were included. Data such as hypertension, serum creatinine, serum eGFR, cardiac events, mortality, diabetes, quality of life and psychological well-being have prospectively been recorded [37]. The Rotterdam study comprises three different cohorts. We will use the second and the third cohort to select non-donors (*n* = 6943), since these comprises more study parameters compared to the first cohort. Multiple follow-up examinations of these two cohorts were conducted between 2000 and 2012. Given the size of both studies, sufficient data on all study parameters will be available in our reference group.

### Outcome measures

The primary outcome is kidney function in serum creatinine and eGFR (calculated with the CKD-EPI formula) [38]. Secondary outcomes are: diastolic and systolic blood pressure as measured by data scope, incidence of hypertension and incidence of diabetes, incidence of fatal and non-fatal cardiovascular diseases, volume of the remaining kidney in cm^3^ by ultrasound (measured by experienced ultrasonographers), quality of life (measured by EuroQoL and SF-12, that can be extracted from any SF-36 [39, 40]), survival, and psychological well-being (for donors aged 45 years and higher with Hospital Anxiety and Depression Scale (HADS) [41], Center for Epidemiologic Studies Depression Scale (CESD) [42], Pittsburgh Sleep Quality Index (PSQI) [43], and questionnaires on Happiness and Life Satisfaction, and for donors aged 44 years and lower with the Beck Depression Inventory (BDI)) [44]. These outcomes will be compared between groups, but also within the donor cohort.

### Missing data and matching

Participants from the Rotterdam Study and SHIP will be restricted from analysis if they would not qualify for live kidney donation based on the following criteria: prevalent diabetes, an eGFR < 60 ml/min/1.73m^2^, and a BMI > 40 (kg/m^2^). To account for the fact that data for covariates at baseline are not complete for all subjects, a multiple imputation approach will be utilized based on the method of chained equations [45]. Using this procedure 10 complete data sets will be created, and for each of the data sets the following steps will be performed: First, donors and non-donors will be 1:4 matched using propensity score matching based on the baseline covariates: age, gender, year of donation/inclusion, BMI, ethnicity, kidney function, blood pressure, pre-existing co-morbidity, glucose level, smoking, alcohol use and highest education degree. Exact matching will be required for gender, ethnicity, existing co-morbidity, and smoking. Progressive radius matching will be performed for age, BMI, kidney function and blood pressure. Each non-donor subject will be allowed to serve as a potential match to more than one donor. If the available data in the control group is insufficient, the target ratio of 1:4 donor:non-donor will be reduced accordingly.

### Statistical analysis

All analyses will be performed for each of the completed data sets and the results will be appropriately pooled. For the medical outcomes a conditional linear or Cox regression analysis will be chosen as appropriate for the outcome. For the Cox regression analysis, the proportional hazards assumption will be tested with Schoenfeld residuals. For the psychological outcomes linear mixed-effects models will be used.

For donor subgroup analysis, parametric and non-parametric tests will be chosen as appropriate for descriptive comparisons, Cox regression analysis will be chosen to investigate different outcomes, and mixed models will be chosen for repeated variables, e.g. kidney function and quality of life.

## Discussion

Recently, studies have emerged that demonstrated unfavourable outcomes after live kidney donation with regard to the long-term risk of ESRD and increased risk of mortality [13, 14]. Donors are a screened and selected population of healthy individuals and therefore it is of utmost importance to maximize both short-term and long-term donor safety. However, high quality studies on long-term outcome after live kidney donation are not very common. Till date there are nine studies in which long-term outcomes are compared between donors and selected non-donors with a follow-up of ten or more years. These studies demonstrate contradictory results regarding the incidence of morbidity and mortality following donation [15–22, 46].

There are differences in the methodology of these studies questioning the comparability of donors and the selected non-donors. No other study has looked into the long-term consequences of live kidney donation in the Dutch health care system. The nine studies were published between 1992 and 2015, with the number of donors ranging from 30 to 2269 and the average follow-up period ranging from 10.9 to 23.7 years. Also, most studies report on a selection of their entire donor cohort. We intend to include our consecutive cohort from the conception of our live kidney donor program with an average follow-up of ten years. Furthermore, due to the follow-up study visits next to registry data, we will have the current physical examination, laboratory results, and medical status. This will provide additional information on potential donors at risk for unfavourable outcomes. The previous studies derived their non-donors from siblings of the donors [17], background/general population [16, 21, 22], and population-based cohort studies [15, 18, 19, 46]. Furthermore, the matching of donors to non-donors was performed based on different characteristics, e.g. age, gender, BMI. Due to our extensive data collection of donors and the prospective data collection of our comparison group we can strive for more comparability between donors and non-donors. We intend to match our donors on all characteristics provided in previous studies and more for a better comparability of donors and non-donors.

All these variations might explain the contradictory results. As donors are a highly selected healthy population, comparison to the general population might lead to an underestimation of long-term risks [16]. For this reason, a study on long-term outcome, including baseline data and a well-matched non-donor group, is the design of choice. In the design of our study we tried to adhere to suggestions of authors of these previous studies. The current donor-cohort of our study forces us to use two different population-based cohort studies to form our non-donor group, as no single population-based study incorporating representative individuals with a broad enough age range provided the desired set of parameters. Nevertheless, this design will facilitate in a one to four proportion. Previous studies in our center in the same living donor population have always resulted in a high response [5, 6, 27, 47]. Although a selection bias can never be precluded, analysis of the non-responding group should provide us with an indication of whether the sample is representative. Our study population includes elderly donors and donors with minor co-morbidity. As there is currently little research on these sub-groups, this study should clarify the ambiguities concerning the long-term outcomes in these groups.

With this study we will try to overcome the limitations of previous studies. We aim to assess the long-term consequences of live kidney donation after a decade post-donation. This study will compare donors with a matched group of non-donors, regarding kidney function, kidney-related comorbidity, quality of life and psychological outcomes.
